# Changes in Physical Activity, Healthy Diet, and Sleeping Time during the COVID-19 Pandemic in South Korea

**DOI:** 10.3390/nu14050960

**Published:** 2022-02-24

**Authors:** Hyukjin Mun, Eun Sun So

**Affiliations:** 1 School of Nursing, Hanyang University, Seoul 04763, Korea; antkwkror@naver.com; 2 College of Nursing, Jeonbuk National University, Jeonju-si 54896, Korea

**Keywords:** COVID-19, health behavior, physical inactivity, unhealthy diet, sleep time, South Korea

## Abstract

The coronavirus disease 2019 (COVID-19) pandemic and subsequent social distancing orders may have changed health behaviors adversely. This study aims to examine changes in physical activity, diet, and sleep patterns during the pandemic in South Korea and to identify the factors influencing adverse changes in these behavioral indicators. Data from the Community Health Survey conducted in 2020 with a total of 229,269 adults were used, employing multivariate logistic regression and a classification and regression tree model. Participants reported decreased physical activity (49.6%), an increase in unhealthy diet (17.0%), and decreased sleep time (9.4%). Changes in adverse health behaviors were significantly related to being female, being in poor subjective health, not having hypertension or diabetes, engaging in other unhealthy behaviors, and complying with COVID-19 prevention guidelines. While those with adverse physical activity and unhealthy diet changes were younger and concerned about COVID-19 infection, the participants with adverse sleep changes were older, experienced economic stress (unemployed or recipients of basic living benefits), and had other unhealthy behaviors (obesity, severe stress, current smoking, and binge alcohol consumption). Public health efforts to intervene in these adverse health behaviors during the COVID-19 pandemic should target the variables shown to be significant in this study.

## 1. Introduction

The coronavirus disease 2019 (COVID-19) pandemic, caused by severe acute respiratory syndrome coronavirus 2 (SARS-CoV-2), has created an unprecedented crisis worldwide [1]. To combat the disease, most countries have implemented quarantine policies such as physical distancing and other orders to mitigate transmission [2]. In South Korea (hereafter, Korea), the government enacted social distancing orders, with four levels of restriction based on the regional severity of disease spread. These orders generally included guidelines to limit the number of people in private gatherings, prohibit large-scale events, restrict access to public facilities, close schools, limit business hours at night, and to define quarantine orders [3].

Health behaviors are critical factors in cardiovascular disease, along with related risk factors such as obesity, hypertension, and diabetes, and may even lead to death [4,5,6]. Physical activity, a healthy diet, and sufficient sleep have proven associations with cardiovascular disease prevention [4,7,8]. However, during the pandemic, due to the COVID-19 disease itself and the related quarantine policies, the quality of health behaviors has deteriorated in many countries worldwide [9,10,11]. The negative behaviors include decreased physical activity, increased unhealthy food consumption, and increased sleep time [8,9]. In Korea, before the COVID-19 epidemic, the government conducted nationwide education initiatives to promote the importance of health-related behaviors for the management of chronic diseases. In the 2019 Korea National Health and Nutrition Examination Survey, 52.6% of men and 42.7% of women responded that they exercised regularly, while 15.4% of adults stated that they consumed unhealthy food such as delivery food or fast food, and adults responded they slept 6.85 h on average [12]. However, studies of the Korean population by the Korean Society for the Study of Obesity (KOSSO) [10,13,14] have demonstrated that physical activity has decreased and that unhealthy foods such as delivered foods and snacks, as well as irregular eating and sleep times, have increased during the pandemic compared to the pre-COVID-19 period. The KOSSO study also reported that 46% of Koreans had gained over 3 kg and that a majority had gained some weight. Because of the relationship of health behaviors with obesity and cardiovascular disease [4,8], changes in adverse health behaviors have been emphasized.

Various factors influence compliance with recommended health behaviors, including personal demographics, health status, other health behavior characteristics, and environmental characteristics such as the COVID-19 pandemic [5,15]. However, changes in adverse health behaviors and the characteristics influencing them have not been fully analyzed, as previous studies have had limitations in terms of the study samples and variables considered [9,11,15,16]. Not all these studies used nationally representative materials, and no previous studies included variables related to COVID-19, such as suspected COVID-19 symptoms, compliance with COVID-19 prevention guidelines, and concerns about COVID-19 infection. In the current situation, there is a need for studies about generalizable health behaviors that reflect the characteristics of COVID-19 and current political circumstances [17]. Understanding how the COVID-19 pandemic adversely affects health behaviors will help public health professionals better cope with the negative health effects of COVID-19.

The objective of this study is to examine the adverse changes in physical activity, diet, and sleep and to identify the factors (such as demographic characteristics, health status, other health behaviors, and COVID-19 pandemic-specific characteristics) influencing these changes since the COVID-19 pandemic began and the subsequent social distancing orders were implemented.

## 2. Materials and Methods

### 2.1. Study Data and Study Design

The present study used data from the 2020 Community Health Survey (CHS), collected by the Korea Disease Control and Prevention Agency (KDCA) [18]. The CHS, a cross-sectional study, has been conducted annually to evaluate local residents’ health status and identify the reasons for local health problems by establishing and assessing regional healthcare plans and to compare regional medical gaps and develop local health services by producing consistent local health statistics through a standardized survey system. In addition, it aims to reproduce and establish the regional, evidence-based healthcare business project and its integrated evaluation index of regional local governments. The survey was conducted in cooperation with 17 province governments, 255 regional community health centers, and 34 universities, and the operating committee, which comprised a local government committee, a university committee, and a professional subcommittee, reviewed and deliberated the validation of the project. The survey items related to COVID-19 were newly added to the study in 2020.

To obtain a representative adult population aged 19 years and above, two stages of probability proportional to size sampling and systematic sampling were used according to the region and the regional households. The data collection was conducted by trained investigators who visited selected households and assisted participants with the completion of an online questionnaire. The CHS data are available to everyone under the government’s permission after the submission of a letter describing the planned research. The study’s plan letter. We received permission to use the data, and the KDCA offered the raw CHS data. A total of 229,269 people responded to the survey in 2020, and their data were analyzed in this study. The investigation proceeded from 16 August 2020 to 31 October 2020, and the results of computer-assisted personal interviews were analyzed. During the investigation, the social distancing order was at level 2 from 16 August to 13 September, level 2.5 from 14 September to 11 October, and level 1 from 12 to 31 October.

### 2.2. Variable Measurements

Changes in health behaviors, such as physical activity, diet, and sleep time were self-reported by participants as increased, the same, or decreased during COVID-19 pandemic. Compared with before the COVID-19 pandemic, participants were asked whether there had been changes in their amount of exercise, walking, and combined indoor and/or outdoor activities. For sleep, respondents were asked whether their amount of sleeping hours had changed compared with before the COVID-19 pandemic. For unhealthy dietary choices, they were asked whether there had been any changes in the frequency of unhealthy food intake, the amount of fast-food intake, and sugary soda consumption. For all items, the answer choices were ‘increased’, ‘stayed the same’, ‘decreased’, and ‘not applicable’. This study merged responses of ‘not applicable’ with ‘stayed the same’, since the ‘not applicable’ choice was for respondents who did not engage in a specific health behavior (e.g., regular exercise). Only sleep time did not have the ‘not applicable’ choice.

Independent variables included demographics such as age, sex, household income (recipient of basic living benefits: yes or no), education (uneducated (no schooling or attended a Korean traditional village school), high school and less, or university and above), and employment (yes or no).

Health status variables included (1) subjective health status (bad = very bad, bad, or fair; good = good and very good), (2) obesity (based on body mass index (BMI) calculated by self-reported height and weight, with obese for BMI > 25 kg/m^2^, overweight for 23 to 24.9 kg/m^2^, normal for 18.5 to 22.9 kg/m^2^, and underweight for <18.5 kg/m^2^), and (3) presence of hypertension or diabetes (self-reported: whether diagnosed by a medical doctor or not).

Health behaviors included (1) stress (self-reported based on subjective perceptions: respondents were asked how much stress they felt in daily life, with answer choices of ‘severe’, ‘average’, and ‘none’), (2) smoking (current, past, and never), and (3) binge alcohol consumption (based on the frequency of binge drinking: never (no drinking or no binge drinking), sometimes (once a month or less), and frequently (once a week or more) consuming at least seven glasses of soju (five cans of beer) or five glasses of soju (three cans of beer) for men and women, respectively).

COVID-19 pandemic-specific variables included the presence of suspected COVID-19 symptoms, compliance with COVID-19 prevention guidelines, and concerns about COVID-19 infection. The suspected COVID-19 symptoms included having a cough or fever in the past 3 months. Compliance with COVID-19 prevention guidelines was categorized by ‘yes’ or ‘no’ answers to six questions evaluating whether participants stayed home when sick, covered their mouth and nose when coughing, periodically ventilated their home or workplace, regularly sanitized their home or workplace, wore a mask while away from home, and complied with social distancing. Concerns about COVID-19 infection were assessed by ‘yes’ or ‘no’ answers to five questions, including whether participants were worried about being infected with COVID-19; dying if infected with COVID-19; being blamed or harmed by other people if infected with COVID-19; infecting vulnerable people such as seniors, children, or patients; and possible economic harm (including job loss or difficulty finding work) because of COVID-19.

### 2.3. Data Analysis

Based on the complex survey design, the data were weighted by proportion to represent the total Korean population. Descriptive statistics, the chi-square test, and the t-test were used to investigate participants’ demographic characteristics, health status, health behaviors, COVID-19 pandemic-specific characteristics, and changes in health behaviors, as well as to compare the relationship of various characteristics with changes in health behaviors. Multivariate logistic regression was used to analyze the effects of demographic characteristics, health status, health behaviors, and COVID-19 pandemic-specific factors on increased or decreased health behaviors, respectively. The response ‘stayed the same’ was used as the reference category for health behaviors, as represented by odds ratios (ORs) with 95% CIs. A classification and regression tree (CART) model was used to predict the occurrence of adverse health behaviors, including decreased physical activity, increased junk food ingestion, and decreased sleep time, by dividing the individuals into subgroups based on variables that exhibited significant differences. All statistical analyses, including the analysis of complex survey data, were performed using SPSS version 27 (IBM, Armonk, NY, USA).

### 2.4. Ethical Considerations

The KDCA offered the raw CHS data after submission of the study’s purpose and analysis plan letter, and we got permission to use the data on 25 August 2021. Statistics Korea approved the CHS protocol (approval no. 117075). All participants consented to participate in this survey, and all contents in this data were anonymized by deleting information that may identify a particular individual, corporation, or organization.

## 3. Results

### 3.1. Participant Demographics, Health Status, Health Behaviors, and COVID-19 Pandemic-Specific Characteristics in Relation to Changes in Health Behaviors

As shown in Table 1, the study participants were on average 54.53 (±17.78) years of age, were evenly distributed between men and women, were rarely recipients of basic living benefits (3.4%), predominantly had a high school education or less (46.8%) and university or higher education (50.8%), and were employed (61.9%). As for health status, they tended to be in good subjective health (52.7%) and overweight or obese (55.0%), and the most common reported diseases were hypertension (21.1%) and diabetes (9.0%). Considering health behaviors, substantial proportions reported having stress (25.6%), currently smoking (18.2%), and being binge drinkers (49.3%). As for COVID-19 pandemic-specific characteristics, only 1.4% had suspected COVID-19 symptoms, and the majority of participants complied with COVID-19 prevention guidelines (78.7%) and had concerns about COVID-19 infection (78.5%).

The health behaviors affected by the COVID-19 pandemic are presented in Table 2. Almost half of the participants (49.6%) reported decreased physical activity, whereas 5.7% stated that their level of physical activity had increased. Furthermore, 17.0% reported an increase in unhealthy eating, whereas 8.4% stated that their unhealthy eating had decreased. For sleep, the responses were 9.4% for decreased sleep time versus 12.0% for increased sleep time.

### 3.2. Influence of Participant Characteristics on Changes in Health Behaviors 

Table 3 and Table 4 present the relationships of various participant characteristics to changes in health behaviors and the degree to which they influenced those changes. All participant characteristics showed statistically significant relationships with changes in health behaviors. Significant relationships with decreased physical activity were found for demographic characteristics, subjective health status, health behaviors, and all COVID-19 pandemic-specific characteristics, with the exception of household income and suspected COVID-19 symptoms. When compared to those whose physical activity level stayed the same, those with decreased physical activity were more likely to be younger, female, more educated, unemployed, and in poor or fair subjective health. They were also more likely to be normal weight, overweight, or obese than underweight; less likely to have hypertension and diabetes; more likely to have severe or average stress; less likely to be current smokers but more likely to be ex-smokers; more likely to binge drink; and more likely to comply with COVID-19 prevention guidelines and to have concerns about COVID-19 infection. Participants with decreased physical activity were significantly more likely to report a worse subjective health status, were less likely to have hypertension and diabetes, were more likely to have stress, and were more likely to engage in binge alcohol consumption than those with increased physical activity. 

Significant associations with consuming a more unhealthy diet were found for all demographic characteristics, subjective health status, health behaviors, and COVID-19 pandemic-specific characteristics, except for household income, employment, and suspected COVID-19 symptoms. When compared to those whose diet stayed the same, those with a more unhealthy diet were more likely to be younger, female, more educated, with a poor or fair subjective health status. They were also more likely to be obese, overweight, or normal weight than underweight; less likely to have hypertension and diabetes; and more likely to have severe or average stress, be a current or ex-smoker, binge drink, comply with COVID-19 prevention guidelines, and be concerned about COVID-19 infection. Furthermore, when compared to those with an increase in healthy eating, those with a more unhealthy diet were significantly more likely to be female, have a higher level of education, and have a worse subjective health status. They were also less likely to report hypertension and diabetes but more likely to experience stress, be a current smoker, sometimes binge drink, and be concerned about COVID-19 infection. 

Decreased sleep time showed significant relationships with all demographic characteristics, health status (except the presence of diabetes), health behaviors (except sometimes binge drinking), and COVID-19 pandemic-specific characteristics (except suspected COVID-19 symptoms). Compared to those whose sleep stayed the same, those with decreased sleep time were more likely to be older, female, recipients of basic living benefits, more educated, unemployed, in poor or fair subjective health, and obese or overweight rather than underweight. Furthermore, they were less likely to have hypertension and more likely to have severe or average stress, be a current or ex-smoker, binge drink frequently, and comply with COVID-19 prevention guidelines. Moreover, compared to those with increased sleep time, those with decreased sleep time were significantly more likely to be obese or overweight, less likely to report hypertension, and more likely to have severe stress and be compliant with COVID-19 prevention guidelines.

**Figure 1:**
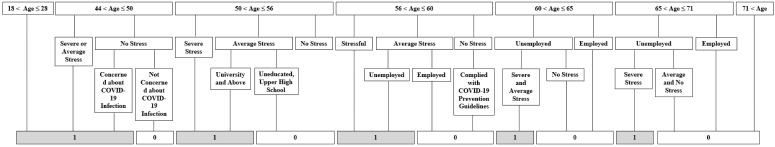
Classification and regression tree (CART) model to predict the occurrence of adverse health behaviors. 0, positive changes in health behaviors. 1, adverse changes in health behaviors.

As Figure 1, CART analysis was used to predict the occurrence of adverse health behaviors by dividing the individuals into subgroups. Age was identified as the best discriminator; whereas adults aged 44 years and younger presented adverse changes in one or more health behaviors, those aged 72 years and older showed positive changes. The second nodes were stress and employment and the third nodes were concerns about COVID-19 infection, education, employment, and stress. Based on this analysis, those at risk for adverse changes in health behaviors were (1) those aged 45–50 years with severe or average levels of stress and those aged 45–50 years with no stress who were concerned about COVID-19 infection, (2) those aged 51–56 years with severe stress and those aged 51–56 years with average stress who had a university and above education, (3) those aged 57–60 years with severe stress and those aged 57–60 with average stress who were unemployed, (4) those aged 61–65 years with unemployment who had severe or average stress, and (5) those aged 66–71 years who were unemployed and had severe stress.

## 4. Discussion

This study demonstrated changes in physical activity, diet, and sleep time that occurred during the COVID-19 pandemic and the subsequent period of social distancing in Korea and identified the factors (i.e., demographic characteristics, health status, other health behaviors, and COVID-19 pandemic-specific characteristics) that influenced these changes among a representative sample of Korean adults. This study found that, although physical activity and unhealthy diets changed in adverse ways, sleep time changed in a positive way. This study found that those with adverse physical activity and unhealthy diet changes were generally younger, female, and in poor subjective health; did not have hypertension or diabetes; had other unhealthy behaviors; were compliant with COVID-19 prevention guidelines; and had concerns about the COVID-19 infection. In contrast, those with adverse sleep time changes were older, female, in poor economic status (recipients of basic living benefits and unemployed), and in poor subjective health; tended not to have hypertension or diabetes; had high levels of unhealthy behaviors (obesity, severe stress, currently smoking, and binge drinking frequently); and were compliant with COVID-19 prevention guidelines. Finally, the first to third most important predictors of these adverse health behaviors were identified as age, stress, unemployment, concerns about COVID-19 infection, and education.

The social distancing order limiting the number of people at gatherings, meetings, and events has contributed to decreased physical activity and an increase in unhealthy eating through telecommuting, socially distanced learning, and increased consumption of delivered foods. Likewise, in an analysis of the KDCA conducted in Korea in 2020, compared to before the COVID-19 epidemic, the proportion of individuals who exercised regularly dropped from 52.6% of men and 42.7% of women to 48.3% and 43.0%, respectively, and the proportion of those who consumed unhealthy food (e.g., delivery food or fast food) increased from 15.4% and 35.0% to 18.7% and 38.5%, respectively (data not presented). In addition, previous studies have shown significant differences in how the four levels of the social distancing order influenced the decreased physical activity numbers. Level 2 was stricter than level 1, for example, and recommended curtailing gatherings, meetings, and events, as well as shutting down many facilities (both public and private). Other studies have investigated an increase in unhealthy eating by comparing pre-COVID-19 body weight to weight during the COVID-19 pandemic [9,10,15,19]. Our study also found a relationship between compliance with COVID-19 prevention guidelines and higher odds of weight gain and unhealthy eating. Social distancing orders contribute to increased sleep time by increasing time at home, with flexible work and study hours possibly allowing for more free time [9,10]. Evaluating sleep time is not straightforward. An adequate sleep time for adults is 7–9 h [20], while excessive sleep time (more than 9 h) has been associated with a lower quality of life and decreased productivity in school and the workplace [21,22].

In this study, younger adults were more likely to report decreased physical activity, possibly because they had to stay indoors more than they did pre-COVID-19 [23]. This study differed from previous studies [9,19,23], which found that females were more likely to have decreased physical activity, possibly because they experienced more stress, felt less motivated, and had fewer resources available to them than men did [9]. This result could reflect cultural bias. The influence of low socioeconomic status (i.e., lower income and unemployment) on decreased physical activity is consistent with previous studies [9,24]. Those with no reported disease (hypertension or diabetes) were more likely to exhibit decreased physical activity, since those with chronic diseases tend to engage in healthy behaviors, including physical activity, possibly in light of the widely known fact that physical activity is important for those with chronic diseases [25]. Individuals demonstrating other unhealthy behaviors and feeling that their health status was poor were more likely to report decreased physical activity. This might be explained by the tendency of unhealthy behaviors to promote other unhealthy behaviors [26], particularly behaviors that can potentially lead to a perception that one’s health status is poor [27]. Those with concerns about COVID-19 infection were more likely to report decreased physical activity. Perceived susceptibility and concerns about the severity of the disease can affect individual behavior including compliance with COVID-19 prevention guidelines [28].

In this study, the factors influencing an increase in unhealthy eating were similar to those influencing a decrease in physical activity. However, when comparing those behaviors, despite noting positive characteristics (such as not having hypertension or diabetes, in particular), an unhealthy diet can still become a problem when concerns about COVID-19 infection or stress are high. Those with hypertension and diabetes tended to engage in positive changes to their diet behaviors to overcome other obstacles they faced under the quarantine order. Consumption of processed and fast foods, which are high in sodium and saturated fats, is well known to increase the risk of hypertension and cardiovascular disease. Carbonated drinks, which are high in sugar, contribute to abdominal obesity and insulin resistance, increasing the risk of type 2 diabetes and cardiovascular disease [29]. The increase in adverse health behaviors caused by the COVID-19 pandemic and the potential long-term effects of those unhealthy behaviors must be given serious attention.

This study has several limitations. First, the survey was conducted through self-reporting; the data may lead to an under- or overestimation. Second, this study investigated qualitative assessments of health behaviors. Further studies using valid tools for measuring variables quantitatively are needed. However, a strength of this study is that its findings are nationally representative.

Much like the benign association between the absence of disease and unhealthy behaviors such as decreased physical activity and unhealthy eating, concerns about COVID-19 infection did not affect changes in sleep, whereas severe stress and severe levels of other unhealthy behaviors were strongly associated with a decrease in sleep time in this study. It is likely that members of socioeconomically vulnerable groups, including those who were older, women, recipients of basic living benefits, and unemployed [9,16], experienced decreased sleep time due to economic stress rather than to health concerns about COVID-19 infection or chronic diseases [9,30]. The increased unemployment rate in Korea and losses of income during COVID-19 (in 2020) [31], as well as other financial difficulties, create intense stress to a comparable extent to other unhealthy behaviors such as obesity, smoking, and binge drinking, as shown in this study. Since behavior-change coping mechanisms may differ across specific subpopulations [9], our study explored these relationships further through the CART analysis. Therefore, stress management education is suggested as an intervention to help reduce or alleviate the long-term negative health effects of adverse changes in physical activity and diet in healthy people for reasons including fears or health concerns related to COVID-19. Additional interventions to help reduce adverse changes in sleep related to the economic difficulties caused by COVID-19 are also needed.

## 5. Conclusions

This study found that the environment created by the COVID-19 pandemic had adverse effects on physical activity, diet, and sleep. Common denominators for all three adverse health behaviors were being female, having subjective poor health, not having hypertension or diabetes, engaging in other unhealthy behaviors, and complying with COVID-19 prevention guidelines. Those with adverse physical activity and unhealthy diet changes were younger and were concerned about COVID-19 infection. Those with adverse sleep time changes were older, in severe economic conditions (recipients of basic living benefits and unemployed), and had other severe unhealthy behaviors (obesity, severe stress, current smoking, and frequent binge drinking). Public health efforts to intervene in adverse health behaviors during the COVID-19 pandemic should target the variables shown to be significant in this study (e.g., by providing stress management education and financial support).

## Figures and Tables

**Table 1:** Participant demographics, health status, health behaviors, and COVID-19 pandemic-specific characteristics.

Characteristics		Participant, *n* (%)
Total		229,269 (100)
	Mean ± SD	54.53 ± 17.78
Age	19–39 years	51,490 (33.0)
	40–59 years	80,505 (39.0)
	More than 60 years	97,274 (28.0)
Sex	Male	103,894 (49.6)
	Female	125,375 (50.4)
Household income	Basic living benefits	9109 (3.4)
	No basic living benefits	220,109 (96.6)
	Uneducated	12,813 (2.4)
Education	High school and less	129,936 (46.8)
	University and above	86,252 (50.8)
Employment	Yes	138,970 (61.9)
	No	90,236 (38.1)
	Poor	30,903 (9.5)
Subjective health status	Fair	88,391 (37.8)
	Good	109,967 (52.7)
	Obese	68,442 (31.0)
Obesity	Overweight	55,035 (24.0)
	Normal	91,445 (40.8)
	Underweight	9263 (4.1)
Hypertension	Yes	64,022 (21.1)
	No	165,218 (78.9)
Diabetes	Yes	26,839 (9.0)
	No	202,402 (91.0)
	Severe	50,729 (25.6)
Stress	Average	119,962 (54.2)
	No	58,508 (20.2)
	Present	37,405 (18.2)
Smoking	Ex	41,901 (18.6)
	No	149,937 (63.2)
	Frequently	30,266 (17.8)
Binge alcohol consumption	Sometimes	50,975 (31.5)
	No	116,976 (50.7)
Suspected COVID-19 symptoms	Yes	2648 (1.4)
	No	226,620 (98.6)
Compliance with the COVID-19 prevention guidelines	Yes	168,427 (78.7)
	No	60,842 (21.3)
Concerns about COVID-19 infection	Yes	185,041 (78.5)
	No	44,228 (21.5)

COVID-19, coronavirus disease 2019.

**Table 2:** Changes in physical activity, diet, and sleep time during the COVID-19 pandemic in South Korea.

Variables		Participant, *n* (%)
Physical activity	Increased	11,752 (5.7)
	Stayed the same	120,495 (44.8)
	Decreased	96,984 (49.6)
	Increased	27,505 (17.0)
Unhealthy diet	Stayed the same	181,758 (74.6)
	Decreased	19,925 (8.4)
	Increased	23,429 (12.0)
Sleep time	Stayed the same	186,324 (78.6)
	Decreased	19,493 (9.4)

**Table 3:** Relationships of participant characteristics with changes in health behaviors during the COVID-19 pandemic.

		Physical Activity				Unhealthy Diet				Sleep Time			
		Increase	Stayed the Same	Decrease	*p*	Increase	Stayed the Same	Decrease	*p*	Increase	Stayed the Same	Decrease	*p*
Age	Mean ± SD	49.09 ± 0.18	51.08 ± 0.08	46.62 ± 0.07	<0.001	38.12 ± 0.08	51.35 ± 0.06	47.25 ± 0.16	<0.001	43.57 ± 0.14	49.54 ± 0.06	48.84 ± 0.14	<0.001
Sex	Male	5355 (48.9)	57,552 (53.5)	40,976 (46.1)	<0.001	12,139 (47.0)	82,515 (50.1)	9208 (50.3)	<0.001	10,882 (48.5)	86,069 (51.0)	6931 (38.5)	<0.001
	Female	6397 (51.1)	62,943 (46.5)	125,348 (50.4)		15,366 (53.0)	99,243 (49.9)	10,717 (49.7)		12,547 (51.5)	100,255 (49.0)	12,562 (61.5)	
Household income	Basic living benefits	403 (3.0)	5130 (3.8)	3572 (3.1)	<0.001	587 (1.9)	7720 (3.8)	796 (3.6)	<0.001	912 (3.6)	7203 (3.3)	993 (4.6)	<0.001
	No basic living benefits	11,348 (97.0)	115,335 (96.2)	93,394 (96.9)		26,916 (98.1)	173,993 (96.2)	19,127 (96.4)		22,515 (96.4)	179,080 (96.7)	18,494 (95.4)	
Education	Uneducated	214 (0.8)	9507 (3.6)	3077 (1.5)	<0.001	88 (0.1)	11,907 (3.0)	799 (1.7)	<0.001	532 (1.1)	11,584 (2.7)	693 (1.7)	<0.001
	High school and less	6416 (46.4)	74,645 (52.4)	48,856 (41.9)		8746 (28.1)	109,760 (51.1)	11,384 (47.1)		11,875 (42.0)	106,979 (47.3)	11,072 (49.4)	
	University and above	5115 (52.8)	36,168 (44.1)	44,966 (56.6)		18,664 (71.7)	59,843 (45.9)	7730 (51.1)		11,006 (56.9)	67,525 (50.0)	7713 (49.0)	
Employment	Yes	7158 (60.6)	73,400 (62.4)	58,404 (61.6)	<0.001	18,564 (67.3)	108,094 (60.7)	12,279 (62.0)	<0.001	13,265 (56.3)	114,463 (63.2)	11,234 (58.5)	<0.001
	No	4592 (39.4)	47,055 (37.6)	38,560 (38.4)		8936 (32.7)	73,625 (39.3)	7628 (38.0)		10,162 (43.7)	71,817 (36.8)	8243 (41.5)	
Subjective health status	Poor	999 (7.1)	18,678 (10.5)	11,210 (8.8)	<0.001	1882 (6.2)	26,639 (10.3)	2362 (8.9)	<0.001	2511 (8.4)	25,098 (9.1)	3285 (13.8)	<0.001
	Fair	4495 (37.0)	45,874 (37.3)	38,010 (38.3)		10,498 (37.7)	70,216 (37.8)	7647 (37.6)		8732 (36.2)	71,189 (37.3)	8462 (43.9)	
	Good	6258 (55.9)	55,936 (52.2)	47,763 (52.9)		15,125 (56.1)	84,896 (51.9)	9916 (53.5)		12,186 (55.4)	90,031 (53.6)	7744 (42.4)	
Obesity	Obese	3659 (31.0)	34,852 (30.6)	29,925 (31.3)	<0.001	8813 (32.0)	53,023 (30.4)	6585 (33.8)	<0.001	7627 (32.0)	54,808 (30.8)	6002 (31.2)	<0.001
	Overweight	3031 (25.5)	28,805 (24.3)	23,188 (23.6)		5827 (21.1)	44,436 (24.7)	4762 (33.8)		5455 (22.7)	45,104 (24.3)	4472 (23.0)	
	Normal	4600 (40.1)	47,952 (40.7)	38,880 (41.1)		11,426 (42.0)	72,401 (40.8)	7583 (39.2)		9111 (40.5)	74,375 (40.8)	7950 (41.5)	
	Underweight	370 (3.5)	5176 (4.4)	3714 (4.0)		1307 (4.8)	7266 (4.1)	683 (3.5)		999 (4.8)	7434 (4.0)	828 (4.3)	
Hypertension	Yes	2999 (21.7)	38,126 (23.9)	22,877 (18.4)	<0.001	2754 (8.8)	55,867 (23.8)	5372 (21.2)	<0.001	5260 (16.9)	53,849 (21.8)	4902 (20.4)	<0.001
	No	8752 (78.3)	82,351 (76.1)	74,098 (81.6)		24,749 (91.2)	125,867 (76.2)	14,551 (78.8)		18,166 (83.1)	132,454 (78.2)	14,587 (79.6)	
Diabetes	Yes	1294 (9.2)	15,939 (10.5)	9599 (7.7)	<0.001	1121 (3.5)	23,363 (10.2)	2340 (9.7)	<0.001	2380 (7.6)	22,358 (9.2)	2099 (9.1)	<0.001
	No	10,456 (90.8)	104,538 (89.5)	87,379 (92.3)		26,382 (96.5)	158,372 (89.8)	17,584 (90.3)		21,047 (92.4)	163,943 (90.8)	17,393 (90.9)	
Stress	Severe	2527 (24.0)	22,945 (22.2)	25,248 (28.8)	<0.001	9522 (36.1)	36,738 (23.2)	4453 (25.5)	<0.001	5870 (27.5)	37,184 (23.2)	7670 (42.9)	<0.001
	Average	6269 (54.0)	61,368 (53.9)	52,315 (54.5)		14,584 (52.3)	94,493 (54.5)	10,857 (55.2)		12,154 (52.5)	98,860 (55.6)	8941 (45.1)	
	No	2954 (22.0)	36,310 (23.9)	19,408 (16.7)		3397 (11.6)	50,465 (22.3)	4612 (19.3)		5400 (20.0)	50,223 (21.2)	2878 (12.0)	
Smoking	Current	1677 (15.3)	20,706 (20.0)	15,020 (16.9)	<0.001	2396 (20.8)	22,973 (19.4)	16,526 (17.5)	<0.001	7679 (63.9)	76,806 (60.6)	65,423 (65.6)	<0.001
	Ex	2396 (20.8)	22,973 (19.4)	16,526 (17.5)		4056 (15.6)	34,396 (19.4)	3428 (17.5)		4242 (17.4)	34,759 (19.0)	2895 (16.2)	
	No	7679 (63.9)	76,806 (60.6)	65,423 (65.6)		18,288 (65.1)	118,427 (62.7)	13,177 (64.4)		14,853 (63.0)	121,651 (62.9)	13,421 (66.5)	
Binge alcohol consumption	Frequently	1609 (18.2)	15,501 (17.6)	13,151 (18.0)	<0.001	4842 (21.6)	22,945 (17.2)	2465 (15.7)	<0.001	3667 (19.8)	23,887 (17.5)	2710 (18.3)	<0.001
	Sometimes	2655 (30.2)	23,831 (28.7)	24,486 (34.3)		9221 (41.9)	36,864 (29.0)	4882 (33.5)		6413 (36.1)	40,153 (30.9)	4402 (30.8)	
	No	5587 (51.6)	67,174 (53.8)	44,188 (47.7)		8801 (36.6)	98,143 (53.8)	9979 (50.8)		10,164 (44.1)	97,681 (51.7)	9119 (50.8)	
Suspected COVID-19 symptoms	Yes	140 (1.3)	1250 (1.2)	1257 (1.6)	<0.001	525 (2.1)	1888 (1.2)	235 (1.5)	<0.001	366 (1.9)	2003 (1.3)	279 (1.7)	<0.001
	No	11,612 (98.7)	119,244 (98.8)	95,727 (98.4)		26,980 (97.9)	179,869 (98.8)	19,690 (98.5)		23,062 (98.1)	184,321 (98.7)	19,214 (98.3)	
Compliance with the COVID-19 prevention guidelines	Yes	9090 (79.8)	83,570 (76.0)	75,754 (81.1)	<0.001	22,469 (82.5)	130,088 (77.4)	15,826 (82.2)	<0.001	17,781 (79.0)	135,535 (78.5)	15,102 (80.1)	<0.001
	No	2662 (20.2)	36,925 (24.0)	21,230 (18.9)		5036 (17.5)	51,670 (22.6)	4099 (17.8)		5648 (21.0)	50,789 (21.5)	4391 (19.9)	
Concerns about COVID-19 infection	Yes	9104 (75.2)	95,991 (76.6)	79,919 (80.6)	<0.001	22,338 (79.9)	146,268 (78.1)	16,380 (78.7)	<0.001	18,752 (77.6)	150,077 (78.2)	16,197 (81.5)	<0.001
	No	2648 (24.8)	24,504 (23.4)	17,065 (19.4)		5167 (20.1)	35,490 (21.9)	3545 (21.3)		4677 (22.4)	36,247 (21.8)	3296 (18.5)	

COVID-19, coronavirus disease 2019.

**Table 4:** The influence of participant characteristics on changes in health behaviors during the COVID-19 pandemic.

		Physical Activity		Unhealthy Diet		Sleep Time	
		Increase	Decrease	Increase	Decrease	Increase	Decrease
Age		1.00 (1.00–1.01) **	0.99 (0.99–0.99) ***	0.96 (0.96–0.96) ***	0.99 (0.98–0.99) ***	0.98 (0.98–0.98) ***	1.00 (1.00–1.01) ***
Sex	Female vs. male	1.45 (1.34–1.58) ***	1.48 (1.42–1.54) ***	1.45 (1.38–1.53) ***	1.01 (0.95–1.08)	1.30 (1.23–1.37) ***	1.91 (1.78–2.04) ***
Household income	On basic living benefits vs. not	0.99 (0.83–1.19)	0.96 (0.87–1.06)	0.86 (0.74–1.01)	1.08 (0.92–1.26)	1.21 (1.05–1.39) *	1.43 (1.24–1.64) ***
Education	Uneducated vs. university and above	0.20 (0.14–0.30) ***	0.46 (0.40–0.53) ***	0.14 (0.07–0.27) ***	0.78 (0.59–1.03)	0.58 (0.44–0.76) ***	0.50 (0.37–0.67) ***
	High school and less vs. university and above	0.78 (0.73–0.83) ***	0.71 (0.68–0.73) ***	0.71 (0.68–0.74) ***	1.08 (1.02–1.14) *	1.08 (1.03–1.14) **	1.07 (1.01–1.13) *
Employment	Yes vs. no	0.78 (0.73–0.83) ***	0.84 (0.81–0.87) ***	1.03 (0.99–1.08)	0.92 (0.87–0.97) **	0.64 (0.61–0.67) ***	0.80 (0.76–0.85) ***
Subjective health status	Poor vs. good	0.90 (0.79–1.04)	1.14 (1.07–1.22) ***	1.54 (1.41–1.68) ***	1.08 (0.97–1.21)	1.28 (1.15–1.41) ***	1.78 (1.62–1.95) ***
	Fair vs. good	0.94 (0.88–1.00)	1.06 (1.03–1.09) ***	1.19 (1.14–1.24) ***	1.01 (0.96–1.07)	1.02 (0.97–1.06)	1.30 (1.24–1.37) ***
Obesity	Obese vs. underweight	1.35 (1.12–1.61) **	1.55 (1.42–1.68) ***	1.43 (1.30–1.58) ***	1.50 (1.30–1.74) ***	1.00 (0.90–1.11)	1.25 (1.09–1.43) **
	Overweight vs. underweight	1.35 (1.13–1.61) **	1.47 (1.35–1.59) ***	1.27 (1.15–1.40) ***	1.31 (1.13–1.53) ***	0.97 (0.87–1.08)	1.18 (1.03–1.36) *
	Normal vs. underweight	1.20 (1.01–1.43) *	1.34 (1.24–1.45) ***	1.19 (1.08–1.31) ***	1.26 (1.09–1.46) **	0.94 (0.85–1.04)	1.13 (0.99–1.29)
Hypertension	Yes vs. no	1.05 (0.97–1.15)	0.92 (1.88–0.96) ***	0.77 (0.72–0.82) ***	1.08 (1.00–1.16) *	1.07 (1.00–1.14)	0.86 (0.80–0.92) ***
Diabetes	Yes vs. no	1.05 (0.94–1.18)	0.88 (0.83–0.93) ***	0.80 (0.73–0.89) ***	1.15 (1.04–1.27) **	1.06 (0.97–1.16)	0.95 (0.86–1.06)
Stress	Severe vs. no	1.11 (1.01–1.23) *	1.56 (1.49–1.64) ***	1.84 (1.72–1.96) ***	1.03 (0.95–1.12)	0.99 (0.93–1.06)	3.01 (2.76–3.28) ***
	Average vs. no	0.99 (0.92–1.07)	1.26 (1.21–1.31) ***	1.29 (1.22–1.37) ***	0.99 (0.93–1.06)	0.86 (0.82–0.92) ***	1.38 (1.27–1.50) ***
Smoking	Present vs. no	0.86 (0.79–0.94) ***	0.94 (0.91–0.98) **	1.11 (1.05–1.18) ***	0.95 (0.89–1.01)	1.20 (1.13–1.27) ***	1.23 (1.15–1.31) ***
	Ex vs. no	1.22 (1.12–1.32) ***	1.11 (1.07–1.15) ***	1.16 (1.09–1.22) ***	0.93 (0.87–1.00) **	1.18 (1.12–1.25) ***	1.21 (1.13–1.29) ***
Binge alcohol consumption	Frequently vs. no	1.06 (0.97–1.15)	1.09 (1.05–1.14) ***	1.15 (1.09–1.21) ***	0.83 (0.78–0.89) ***	1.19 (1.12–1.25) ***	1.11 (1.04–1.19) **
	Sometimes vs. no	0.99 (0.93–1.06)	1.13 (1.10–1.17) ***	1.17 (1.12–1.22) ***	1.02 (0.97–1.08)	1.15 (1.10–1.20)***	1.03 (0.98–1.09)
Suspected COVID-19 symptoms	Yes vs. no	1.10 (0.86–1.41)	1.08 (0.95–1.22)	1.11 (0.96–1.28)	1.03 (0.84–1.28)	1.30 (1.11–1.52) **	1.15 (0.95–1.38)
Compliance with the COVID-19 prevention guidelines	Yes vs. no	1.11 (1.03–1.20) **	1.19 (1.14–1.23) ***	1.06 (1.01–1.12) **	1.25 (1.17–1.33) ***	0.97 (0.92–1.02)	1.15 (0.95–1.38) *
Concerns about COVID-19 infection	Yes vs. no	0.90 (0.84–0.96) **	1.23 (1.20–1.28) ***	1.23 (1.17–1.29) ***	1.02 (0.96–1.09)	1.02 (0.97–1.07)	1.08 (1.02–1.15)

* *p* < 0.05, ** *p* < 0.01, *** *p* < 0.001. COVID-19, coronavirus disease 2019.

## Data Availability

The datasets used and/or analyzed during the current study are available from the corresponding author on reasonable request.
